# Supplementary material for: Enhancing autophagy by redox regulation extends lifespan in Drosophila
Source: Nat Commun. 2025 Jun 25;16:5379. doi: 10.1038/s41467-025-60603-w (PMC12198390; doi:10.1038/s41467-025-60603-w)
Supplement: Supplementary file 1 — Supplementary Information [file 41467_2025_60603_MOESM1_ESM.pdf]

Supplementary Information

Enhancing autophagy by redox regulation extends lifespan in *Drosophila*

Authors

Claudia Lennicke[1,2], Ivana Bjedov[3], Sebastian Grönke[4], Katja E. Menger[5], Andrew M. James[5], Jorge Iván Castillo-Quan[6], Lucie A. G. van Leeuwen[1,2], Andrea Foley[1,2], Marcela Buricova[1,2], Jennifer Adcott[6], Alex Montoya[1,2], Holger B. Kramer[1,2], Pavel V. Shliaha[1,2], Angela Logan[5], Filipe Cabreiro[2,7], Michael P. Murphy[5], Linda Partridge[4,6]*, Helena M. Cochemé[1,2]*

Affiliations

[1] MRC Laboratory of Medical Sciences (LMS), Du Cane Road, London W12 0HS, UK.
[2] Institute of Clinical Sciences, Imperial College London, Hammersmith Hospital Campus, Du Cane Road, London W12 0HS, UK.
[3] UCL Cancer Institute, 72 Huntley Street, London WC1E 6DD, UK.
[4] Max Planck Institute for Biology of Ageing, Joseph Stelzmann Strasse 9b, 50931 Cologne, Germany.
[5] MRC Mitochondrial Biology Unit, University of Cambridge, Cambridge Biomedical Campus, Hills Road, Cambridge CB2 0XY, UK.
[6] Institute of Healthy Ageing and GEE, University College London, Gower Street, London WC1E 6BT, UK.
[7] CECAD Research Cluster, University of Cologne, Joseph Stelzmann Strasse 26, 50931 Cologne, Germany.

* correspondence: linda.partridge@ucl.ac.uk; helena.cocheme@lms.mrc.ac.uk

Supplementary Figures 1-4

- Supplementary Figure 1: Ubiquitous catalase up-regulation extends lifespan in flies, independently of oxidative stress resistance.
- Supplementary Figure 2: Catalase-mediated lifespan extension requires autophagy.
- Supplementary Figure 3: Catalase flies undergo an oxidising shift in global thiol redox state.
- Supplementary Figure 4: Redox regulation of autophagy via Atg4 Cys102 extends lifespan.

Supplementary Tables 1-2

- Supplementary Table 1: Oligonucleotide primers used for cloning and genotyping of the Atg4a-WT and Atg4a-C102S flies
- Supplementary Table 2: QPCR primers

Supplementary Figure 1

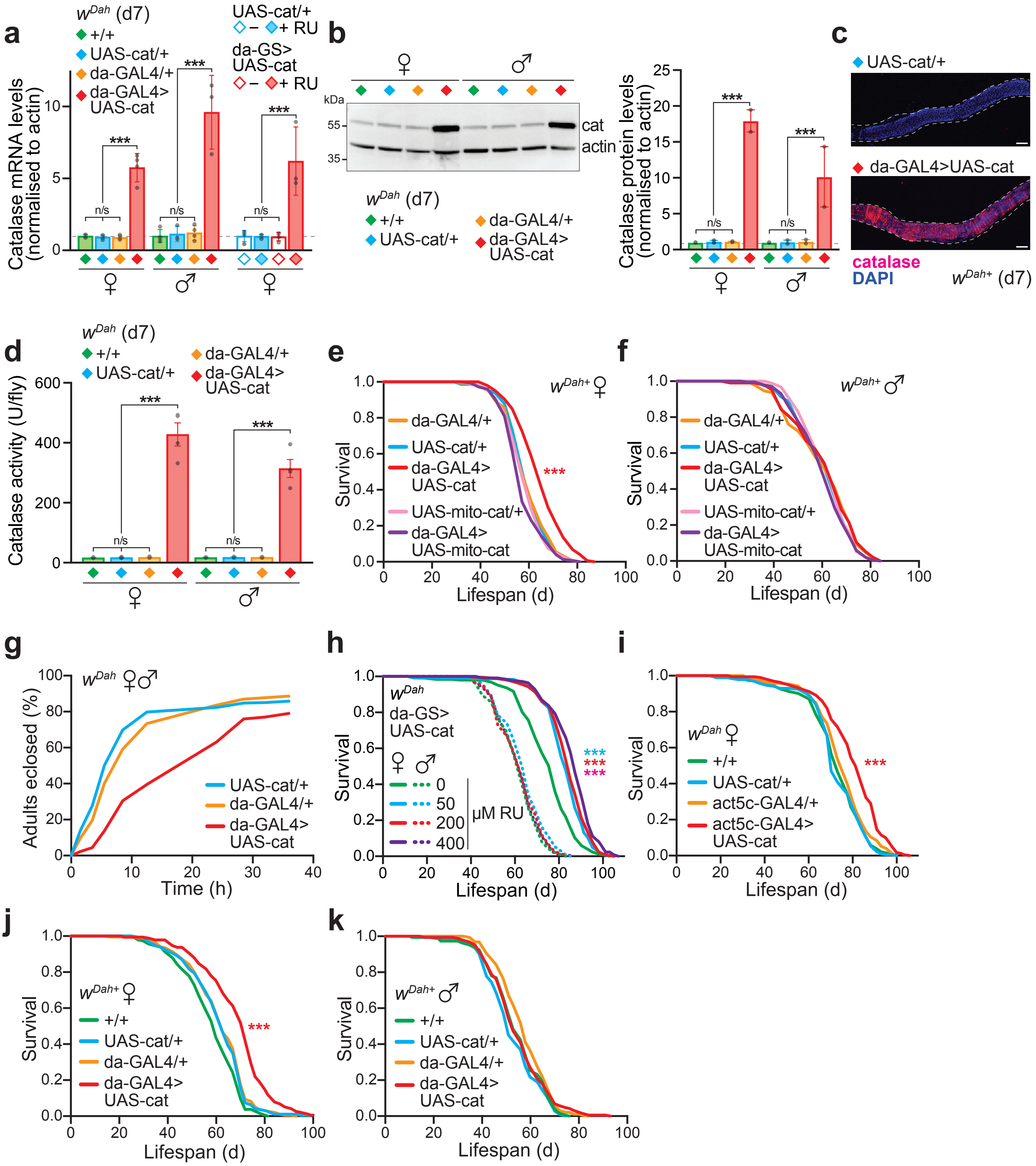

Supplementary Figure 1
(continued)

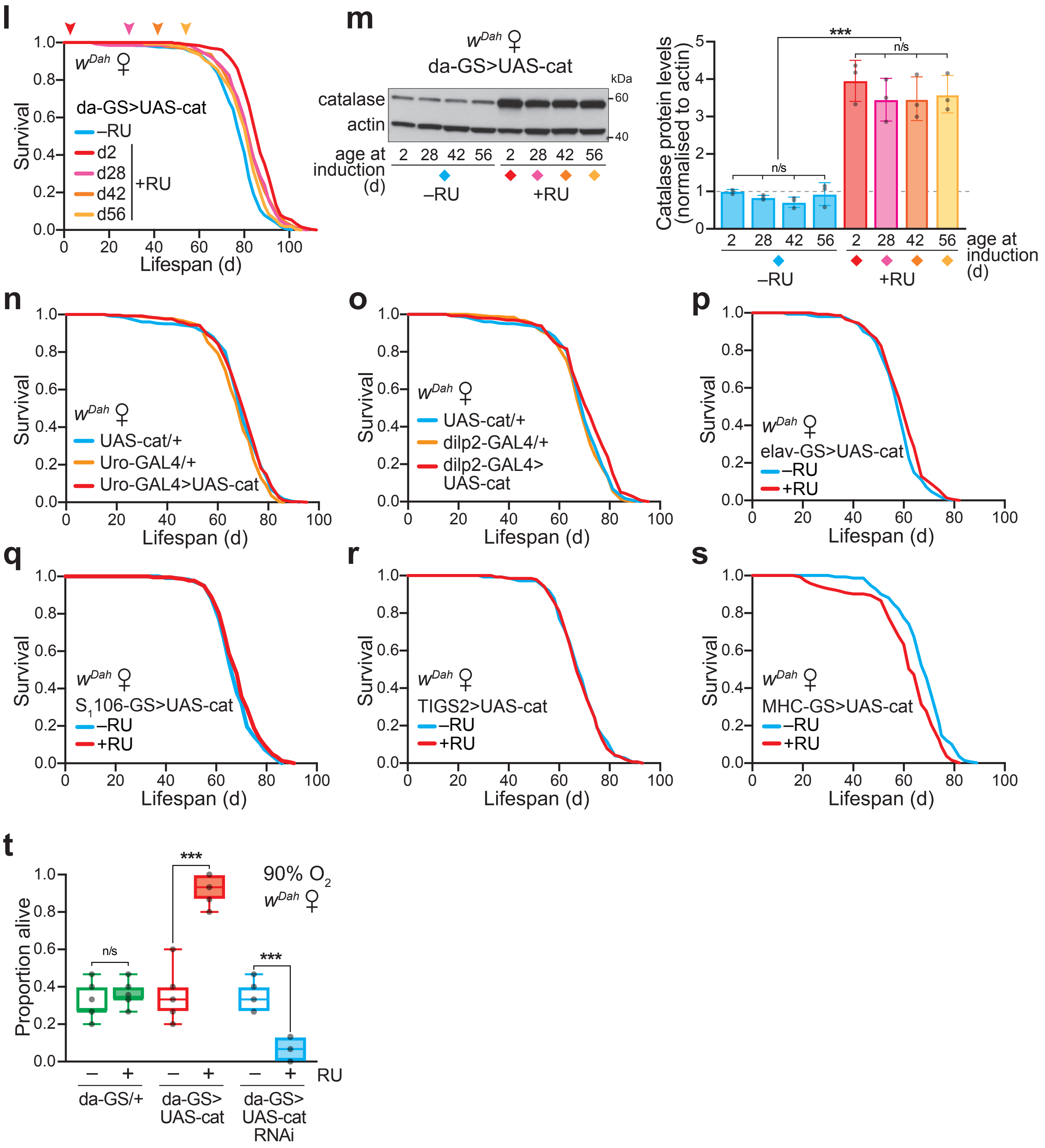

Supplementary Fig. 1 | Ubiquitous catalase up-regulation extends lifespan in flies, independently of oxidative stress resistance. a, Quantification of catalase over-expression by QPCR. Analysis of catalase mRNA levels normalised to actin in constitutive da-GAL4>UAS-cat females and males at d7, and inducible da-GS>UAS-cat +RU females at d9 (=d7 of RU treatment) compared to controls. Data are means ±SD of n=3-4 replicates, each with n=10 whole flies per sample, analysed by one-way ANOVA (Tukey). **b,** Representative Western Blot showing catalase protein abundance in female and male flies at d7. Data are means ±range of n=2 biological replicates, each with n=10 whole flies per sample, analysed by one-way ANOVA (Tukey). **c,** Confocal microscopy of midgut sections showing catalase protein abundance by immunofluorescence (magenta) and nuclei (DAPI, blue) in control (UAS-cat/+) and catalase over-expressor (daGAL4>UAS-cat) females at d7 (scale bar = 100 μm). **d,** Catalase enzyme activity in whole female and male flies at d7. Data are means ±SEM of n=4 replicates per condition, analysed by one-way ANOVA (Tukey). **e-f,** Ubiquitous over-expression of mitochondria-targeted catalase (da-GAL>UAS-mito-cat) does not extend lifespan in females (p>0.05 *v.* UAS-mito-cat/+ and da-GAL4 controls) or males (p>0.05 for all comparisons). Survival assays were performed with n=150 flies per condition. **g,** Development time course. Constitutive ubiquitous catalase over-expression (da-GAL4>UAS-cat) causes a mild developmental delay relative to the UAS-cat/+ and da-GAL4/+ controls. Synchronised L1 larvae (n=500 per genotype) were transferred to SYA vials, and the subsequent eclosion time of adults was recorded. **h,** Dose response of da-GS>UAS-cat lifespan to a range of RU concentrations (0, 50, 200 and 400 μM) in both females and males. Survival assays were performed with n=240-255 flies per condition. **i,** Over-expression of catalase with a different ubiquitous driver extends lifespan (act5c-GAL4>UAS-cat *v.* UAS-cat/+, $p=3.6x10^{-12}$). Survival assays were performed with n=200 females per condition. **j-k,** Lifespan extension by catalase is independent of *Wolbachia* status. Constitutive catalase over-expression (da-GAL4>UAS-cat) extends the lifespan of female flies in a w^{Dah+} background (c), but not males (d) relative to all controls (+/+, UAS-cat/+ and da-GAL4/+). Survival assays were performed with n=200 flies per condition. **l,** Late onset over-expression of catalase using the inducible GeneSwitch system from either middle-age (d28 and d42) and old-age (d56) extends the lifespan of female flies. Lifespans were performed with n=270 flies per condition. Original survival curves corresponding to Fig. 1e. **m,** Quantification of catalase protein levels by Western blotting with age and upon late-onset induction (see Fig. 1e). Flies (da-GS>UAS-cat) were sampled 7 d after the indicated age of RU treatment initiation. Left, typical Western blot probed against catalase with actin as a loading control. Right, quantification by densitometry. Data are means ±SD of n=3 biological replicates per time point, each with n=5 flies per sample, analysed by one-way ANOVA (Tukey). **n-s,** Tissue-specific over-expression of catalase: **n,** in the Malphigian tubules (the fly equivalent of the kidney) driven by Uro-GAL4 (n=300 females per condition, except n=285 for UAS-cat/+); **o,** in the insulin producing cells (IPCs) driven by dilp2-GAL4 (n=300 females per condition, except n=285 for UAS-cat/+); **p,** pan-neuronal driven by elav-GS (n=150 females per condition); **q,** in the intestine and fat body (the fly equivalent of the liver/adipose tissue) driven by S_1106-GS (n=225 females per condition); **r,** in the gut driven by TIGS2 (n=150 females per condition); **s,** pan-muscular driven by the myosin heavy chain driver MHC-GS (n=150 females per condition). **t,** Catalase-RNAi flies are sensitive to hyperoxia stress relative to controls. Incubation at 90% O_2 was initiated at d9 (= d7 of RU treatment), and the fraction alive scored after 4 d. Data are presented as box-and-whisker plots (interquartile range, line at median, min/max error bars) with n=7 vials per condition (each with n=15 flies), analysed by one-way ANOVA (Tukey) (da-GS/+ ±RU, p=0.939; da-GS>UAS-cat ±RU, $p<1x10^{-15}$; da-GS>UAS-catRNAi ±RU, $p=7.01x10^{-6}$). All survival assays (**e**,**f**,**h**,**i**,**j**,**k**,**l**,**m**,**n**,**o**,**p**, **q**,**r**,**s**) were analysed by Log-Rank test (see Supplementary Data 1 for full n numbers and p values). n/s, p>0.05; ***, p<0.001. Source data are provided as a Source Data file.

Supplementary Figure 2

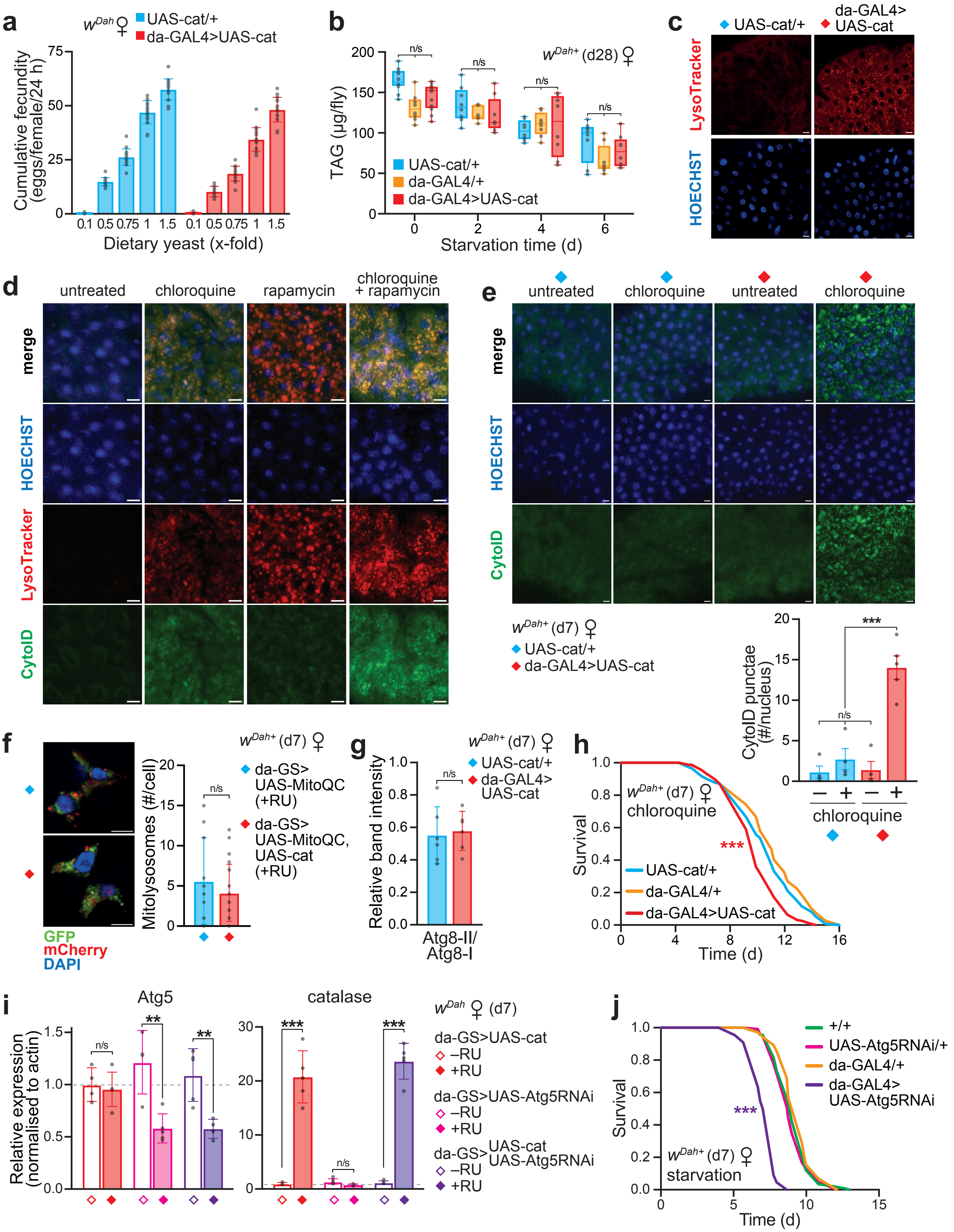

Supplementary Fig. 2 | Catalase-mediated lifespan extension requires autophagy. a, Response of egg laying to DR. Cumulative fecundity (d7+d14+d21) for each genotype at a range of dietary yeast concentrations, with 1x corresponding to standard SYA food. Data are means ±SD of n=16 vials per genotype, each with n=10 females per vial, analysed by one-way ANOVA (Tukey, p<0.001 for all comparisons within each genotype). **b,** Whole body TAG levels in females assayed at d28 (t=0) and in response to a starvation timecourse. Data are presented as box-and-whisker plots (interquartile range, line at median, min/max error bars) of n=4-12 replicates per genotype, each with n=5 females per sample, analysed by two-way ANOVA (Tukey). **c-d,** Confocal imaging of female midguts stained with LysoTracker Red and/or CytoID Green, as indicators of autophagolysosome formation and autophagic flux respectively (scale bar = 10 µm). **c,** Images of individual LysoTracker Red and HOECHST channels, corresponding to Fig. 2f. **d,** Treatment with the autophagy activator rapamycin as a positive control (200 µM from d2) increases LysoTracker Red staining in WT (w^{Dah+}) females, but not CytoID Green signal, indicating that autophagy is induced but not blocked. CytoID Green staining occurs upon co-treatment of rapamycin with the autophagy inhibitor chloroquine (10 mM in SYA, from d7 for 48 h), i.e. when autophagy is induced and blocked. **e,** Autophagy is induced in catalase over-expressors (da-GAL4>UAS-cat) compared to controls (UAS-cat/+), without affecting flux. CytoID staining is apparent in the catalase over-expressors (da-GAL4>UAS-cat) only when treated with the autophagy inhibitor chloroquine (10 mM in SYA, from d7 for 48 h). Data are means ±SEM of n=4-5 replicates per condition, analysed by one-way ANOVA (Tukey). **f**, Catalase up-regulation does not impact mitophagy, as inferred from the mito-QC reporter. Confocal images of dissected midguts showing mitolysosome punctae (scale bar = 5 µm). Data are the means ±SD of n=8-10 counts per genotype, analysed by unpaired two-tailed Student's *t*-test. **g,** Quantification of the Atg8-II/Atg8-I ratio for the Western blot in Fig. 2g. Data are means ±SD of n=6 biological replicates, analysed by unpaired two-tailed Student's *t*-test (p=0.761). **h,** Catalase over-expressor females (da-GAL4>UAS-cat) were sensitive to treatment with the autophagy inhibitor chloroquine (10 mM in sucrose/agar medium) relative to UAS-cat/+ and da-GAL4/+ controls ($p=2.6x10^{-5}$ and $p=3.5x10^{-9}$, respectively). Assays were performed at d7 with n=160 flies per condition. **i,** QPCR quantification of Atg5 and catalase levels upon Atg5 RNAi and catalase over-expression, normalised to actin. Female flies were sampled at d9 (=d7 of RU treatment), using RNA extracted from head and thorax tissue. Data are means ±SD of n=4-5 replicates per condition, each with n=10 flies per sample, analysed by one-way ANOVA (Tukey). **j,** Ubiquitous Atg5 RNAi results in sensitivity to starvation stress. The assay was set up at d7 with n=20-140 flies per genotype, using the GAL4 driver (rather than GeneSwitch) to maintain Atg5-RNAi during starvation stress where RU is not present (p<0.001 for da-GAL4>UAS-Atg5RNAi compared to all controls). All survival assays (**g,i**) were analysed by Log-Rank test (see Supplementary Data 1 for full n numbers and p values). n/s, p>0.05; **, p>0.01; ***, p<0.001. Source data are provided as a Source Data file.

Supplementary Figure 3

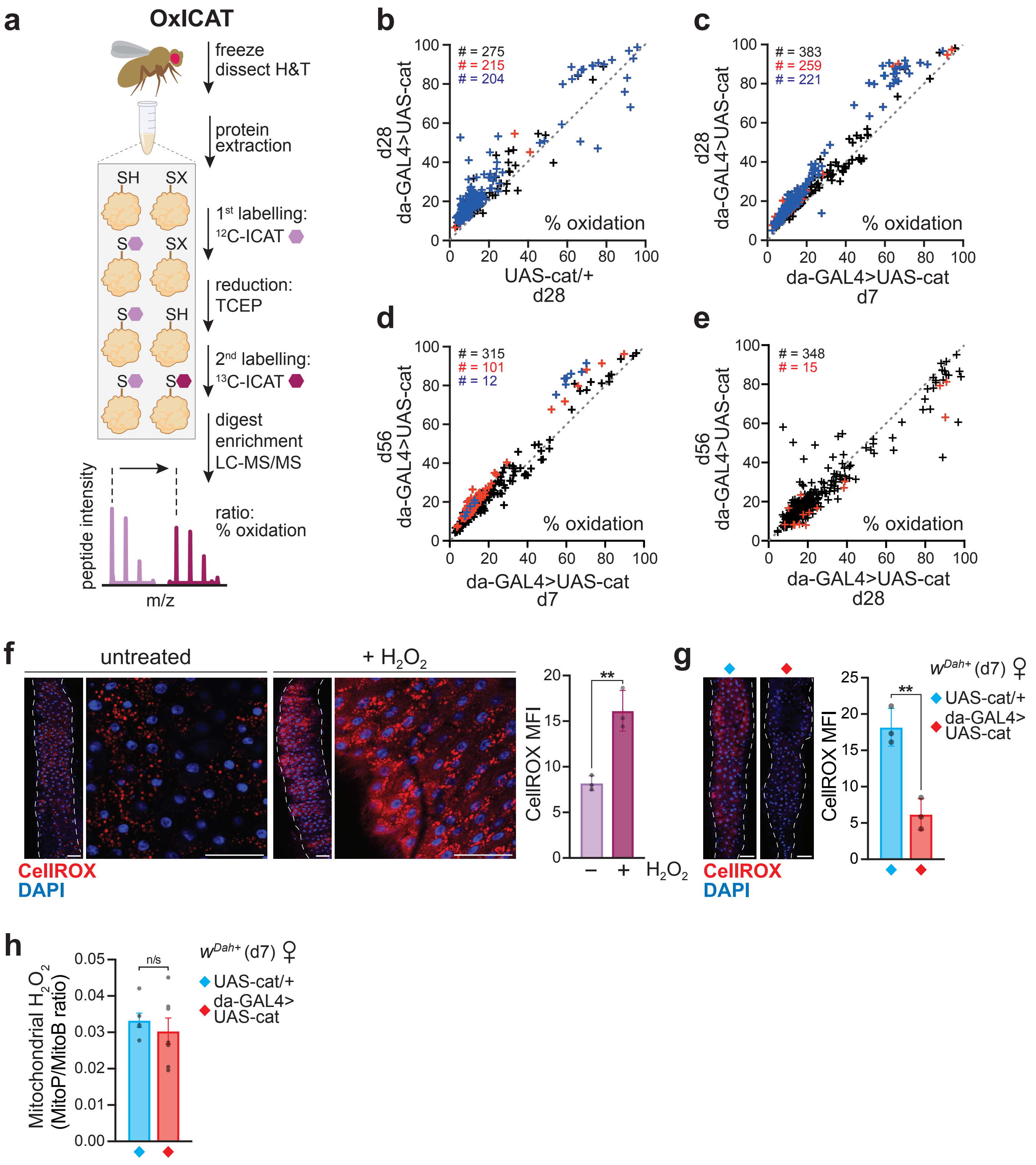

Supplementary Fig. 3 | Catalase flies undergo an oxidising shift in global thiol redox state. a, Scheme of the OxICAT workflow for unbiased redox proteomics. Cysteines are differentially labelled according to redox status using light and heavy ICAT probes. **b-e,** OxICAT analysis. Oxidation state of Cys residues present, comparing control (UAS-cat/+) *v.* catalase over-expressor (da-GAL4>UAS-cat) females at d28 (**a**), d7 *v.* d28 catalase over-expressors (**b**), d7 *v.* d56 catalase over-expressors (**c**), and d28 *v.* d56 catalase over-expressors (**d**). Data points above or below the diagonal dotted line (slope=1) indicate a change in Cys redox state, with red symbols designating low stringency significance ($p<0.05$, assessed by unpaired two-tailed Student's *t*-test) and blue symbols high stringency significance (Benjamini-Hochberg test). The total number of unique Cys-containing peptides is indicated in black. **f-g,** H_2O_2 levels inferred from the fluorescent redox-reactive probe CellROX assessed by confocal microscopy of dissected midguts (scale bar = 50 µm). Data are means ±SD of n=3 samples per condition (MFI, mean fluorescence intensity). **f,** Response of control tissue incubated $\pm H_2O_2$ as a positive control for the assay (p=0.0044). **g,** CellROX staining of catalase up-regulated guts, showing decreased signal compared to control (p=0.003564). **h,** Mitochondrial H_2O_2 levels quantified using the MitoB probe, showing similar levels in control and catalase up-regulated flies. Data are means ±SEM of n=6-7 samples per genotype (p=0.5045). Data (**f-h**) are for d7 females, analysed by unpaired two-tailed Student's *t*-test. n/s, $p>0.05$; **, $p<0.01$. Source data are provided as a Source Data file.

Supplementary Figure 4

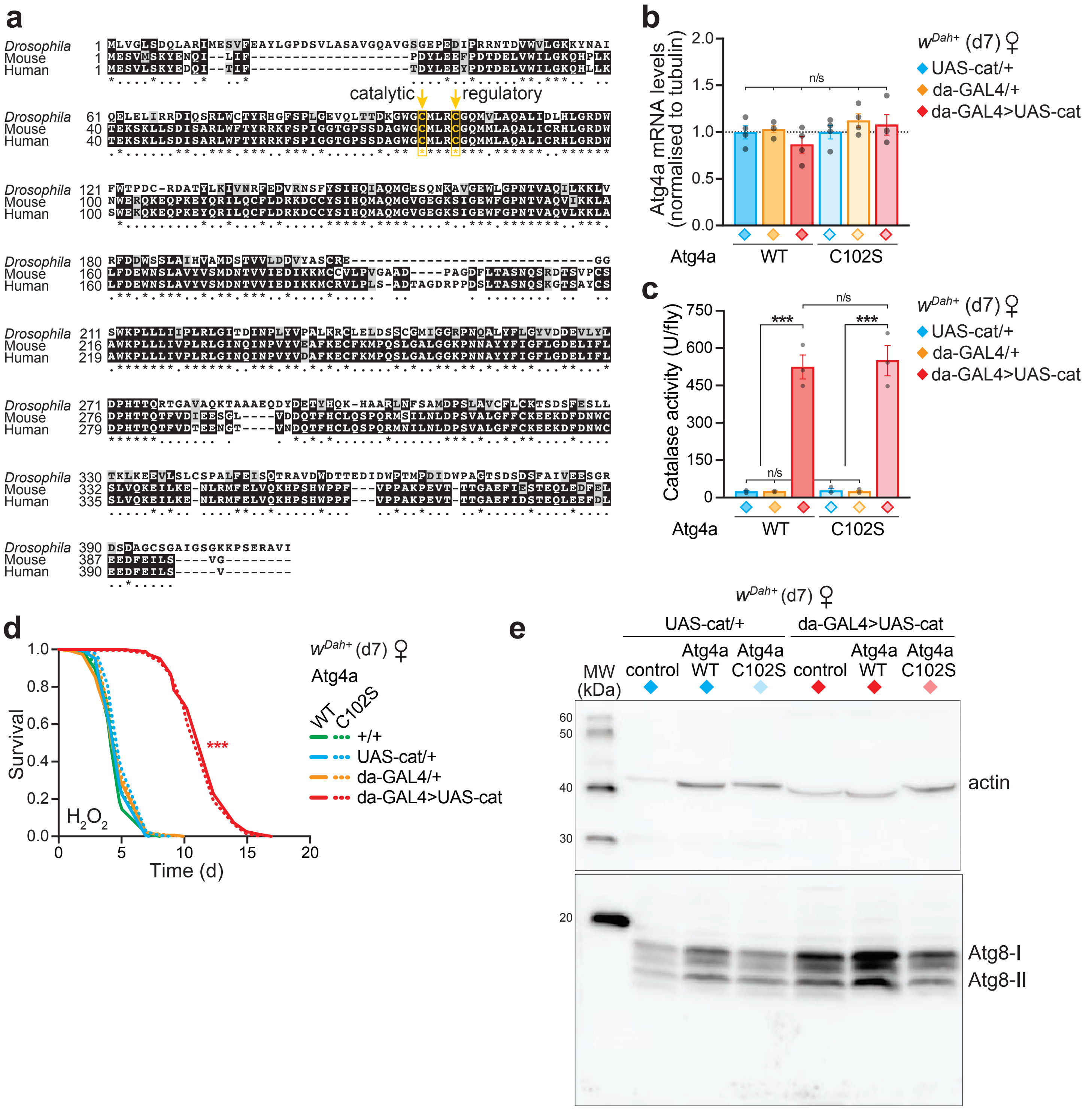

Supplementary Fig. 4 | Redox regulation of autophagy via Atg4 Cys102 extends lifespan. a, Multiple sequence alignment of Atg4a from *Drosophila* with the mouse and human orthologues (UniProt IDs # Q9VPW2, Q8C9S8 and Q8WYN0, respectively) performed in T-Coffee (www.tcoffee.org). **b,** Atg4a expression levels are not affected by the C102S mutation or catalase over-expression, as assessed by QPRC, normalised to tubulin. Data are the means ±SD of n=4 replicates, analysed by one-way ANOVA (Tukey, p>0.05 for all comparisons). **c,** The Atg4a-C102S mutation does not change levels of catalase up-regulation, as measured by enzyme activity. Data are the means ±SD of n=3 replicates, analysed by one-way ANOVA (Tukey). **d,** H_2O_2 stress resistance is comparable between Atg4a-WT flies and Atg4a-C102S knock-in mutants, both under control conditions and upon catalase up-regulation. H_2O_2 treatment (5% v/v in sucrose/agar medium) was initiated at d7, with n=160 females per genotype. Survival assays were analysed by Log-Rank test (see Supplementary Data 1 for full n numbers and p values). **e,** Full Western blots corresponding to data in Figs. 2g and 4c. After transfer, the upper portion of the membrane was probed for actin as a loading control, while the lower portion of the membrane was probed against Atg8. **f,** Autophagy induction in response to catalase over-expression is maintained in d7 Atg4a-WT female flies, but abolished in the Atg4a-C102S mutant background, as assessed by LysoTracker Red staining of midguts (scale bar = 10 μm). Images of individual channels, corresponding to Fig. 4d. **g,** Climbing assay of control (da-GAL/+) and catalase over-expressors (da-GAL>UAS-cat) in Atg4a-WT and Atg4a-C102S backgrounds. Data are presented as box-and-whisker plots (interquartile range, line at median, min/max error bars) of n=5 replicates per condition, each with n=15 flies per sample, analysed by two-way ANOVA (Tukey). **h,** The Atg4a-C102S mutation does not alter egg laying upon catalase up-regulation compared to the control Atg4a-WT background in d7 females. Data are the means ±SEM of n=7 vials per genotype, each set up with n=15 females per vial, analysed by one-way ANOVA (Tukey). n/s, p>0.05; *, p<0.05; **, p<0.01; ***, p<0.001. Source data are provided as a Source Data file.

Supplementary Table 1 | Oligonucleotide primers used for cloning and genotyping of the Atg4a-WT and Atg4a-C102S flies
Lennicke *et al.*

Primer	Sequence	Purpose
SOL897	TATATAGGAAAGATATCCGGGTGAACTTCGATTATCGTATAGACGAGCAGGTTTTAGAGCTAGAAATAGCAAG	5´ primer gRNA
SOL898	ATTTTAACTTGCTATTTCTAGCTCTAAAACAATGTAAGTCGATCAGGGCCGACGTTAAATTGAAAATAGGTC	3´ primer gRNA
SOL926	ATGCGGCCGCAGAGCGAGCAGTGAGGGATAAG	5´ primer donor construct + *NotI* site
SOL927	TAGGTACCGTCCCTGCCGCTCTCTTCTGG	3´ primer donor construct + *KpnI* site
SOL928	CGCCCGTCACTGCCAACAAC	sequencing primer donor construct
SOL929	CCACTCGCCGACCGCTTTG	sequencing primer donor construct + 3´ primer for FLAG genotyping
SOL930	CCAGAACCCTTGTCATCGTCATC	sequencing primer donor construct
SOL931	CCTGCAGAAGAAGCGATTGAAGAA	sequencing primer donor construct
SOL932	CAACACCCTATCCCTTTTCAGAGC	sequencing primer donor construct
SOL933	GGTATTGGATGACGTATGTAAGTGTGG	sequencing primer donor construct
SOL934	CCAAGTTGAAGGAGGAGGTGCTC	sequencing primer donor construct
SOL935	CGGCATCCTCGACCATTTCC	sequencing primer donor construct
SOL953	GTCCACCCCCTTCTCCTTCTGC	5´ primer to identify homozygous FLAG-ATG4a flies (179 bp on FLAG, 137 bp on WT)
SOL954	GGCCAACTGCACTAGCGAGGAC	3´ primer to identify homozygous FLAG-ATG4a flies (179 bp on FLAG, 137 bp on WT)
SOL955	GGGTTCTGGAGGTTCTGGACTGG	5´ primer FLAG genotyping

Supplementary Table 2 | QPCR primers
Lennicke *et al.*

Gene	GenBank Accession	Forward (5' - 3')	Reverse (5' - 3')	Reference
actin (Act5c)	NM_167053.2	CACACCAAATCTTACAAAATGTGTGA	AATCCGGCCTTGCACATG	Bjedov *et al.* 2010
Atg4a	NM_134719.4	TTAACCCGCTTTACGTGCCT	CCTGCCGCTCTCTTCAACTA	This study
Atg5	NM_132162.4	GACATCCAACCGCTCTGCGCA	CAGACGATGACTTCACGTACACC	Bjedov *et al.* 2010
Cat	NM_080483.3	GAACTACTTTGCTGAGGTGGA	CATCTTGTCCGGAGAGGGCT	This study
tubulin (alphaTub84B)	NM_057424.4	TGTCGCGTGTGAAACACTTC	AGCAGGCGTTTCCAATCTG	This study
