# Supplementary material for: Enhancing autophagy by redox regulation extends lifespan in Drosophila
Source: Nat Commun. 2025 Jun 25;16:5379. doi: 10.1038/s41467-025-60603-w (PMC12198390; doi:10.1038/s41467-025-60603-w)
Supplement: Supplementary file 2 — Description of Additional Supplementary Files [file 41467_2025_60603_MOESM2_ESM.docx]

Description of Additional Supplementary Files

**File Name: Supplementary Data 1**

**Description:** Summary of information on the experimental set-up for all survival data in the study (lifespans and stress assays), including exact n numbers, and statistical analysis (p values, Log-Rank test).

**File Name: Supplementary Data 2**

**Description:** Full list of cysteine residues identified in this study by OxICAT redox proteomics, ordered by Uniprot ID. Mean % Cys oxidation ±SD was calculated for control (UAS-cat/+) and catalase over-expressor (da-GAL4>UAS-cat) female flies, at the 3 indicated ages (d7, d28 and d56). Samples consisted of combined head and thorax homogenates, with abdomens removed. Values are reported for cysteine residues detected in at least 3 out of 5 biological replicates (exact n numbers are indicated).

**File Name: Supplementary Data 3**

**Description:** Molecular function and pathway analysis of proteins identified in this study by OxICAT redox proteomics. Gene ontology (GOTERM_MF_DIRECT, Molecular Function, Direct) and pathway (KEGG_PATHWAY) analysis are provided for the proteins listed in Supplementary Data 2. Functional annotations were obtained from DAVID (<https://david.ncifcrf.gov/tools.jsp>).
