# Supplementary material for: Enhancing autophagy by redox regulation extends lifespan in Drosophila
Source: Nat Commun. 2025 Jun 25;16:5379. doi: 10.1038/s41467-025-60603-w (PMC12198390; doi:10.1038/s41467-025-60603-w)
Supplement: Supplementary file 3 — Supplementary Data 1 [file 41467_2025_60603_MOESM3_ESM.pdf]

Supplementary Data 1 | Summary of survival data

Lennicke *et al.*

	Background	Sex	Genotype	Flies per vial	# vials	Total flies (set-up)	Deaths	Censors	Total flies (actual)	Median (d)	ΔMedian (%)		p-value (Log-Rank test)	
													b	*c*
Fig. 1a	w^{Dah}	female	UAS-cat/+	10	20	200	186	12	198	65.3	0	*a*	0.7504	5.7E-07
			da-GAL4/+	10	20	200	189	11	200	64.6	-1.07	*b*		3.3E-06
			da-GAL4>UAS-cat	10	20	200	159	39	198	69.3	+6.13	*c*		
													e	*f*
	w^{Dah}	male	UAS-cat/+	10	20	200	122	75	197	52.1	0	*d*	0.5654	0.0779
			da-GAL4/+	10	20	200	145	55	200	51.8	-0.58	*e*		0.1925
			da-GAL4>UAS-cat	10	20	200	127	67	194	52.8	+1.34	*f*		

	Background/ Sex	Genotype	Condition	Flies per vial	# vials	Total flies (set-up)	Deaths	Censors	Total flies (actual)	Median (d)	ΔMedian (%)		p-value (Log-Rank test)		
													b	*c*	*d*
Fig. 1b	w^{Dah} female	da-GS/+	–RU (0 µM)	15	15	225	215	2	217	77.3	0	*a*	0.7161	0.0737	1.3E-21
			+RU (200 µM)	15	15	225	224	1	225	76.9	-0.52	*b*		0.1751	1.0E-18
		da-GS>	–RU (0 µM)	15	20	300	295	3	298	77.8	+0.65	*c*			1.1E-16
		UAS-cat	+RU (200 µM)	15	20	300	285	1	286	85.1	+10.09	*d*			

	Background/ Sex	Condition	Genotype	Flies per vial	# vials	Total flies (set-up)	Deaths	Censors	Total flies (actual)	Median (d)	ΔMedian (%)		p-value (Log-Rank test)	
													b	*c*
Fig. 1f	w^{Dah} female	5% H_2O_2	UAS-cat/+	15	8	120	120	0	120	3.150	0	*a*	0.0164	5.0E-61
			da-GAL4/+	15	8	120	114	6	120	3.325	+5.56	*b*		4.9E-62
			da-GAL4>UAS-cat	15	8	120	120	0	120	12.200	+287.30	*c*		
													e	*f*
	w^{Dah} male	5% H_2O_2	UAS-cat/+	15	7	105	105	0	105	3.975	0	*d*	0.8243	3.2E-33
			da-GAL4/+	15	7	105	105	0	105	3.825	-3.77	*e*		4.6E-33
			da-GAL4>UAS-cat	15	5	75	74	1	75	6.625	+66.67	*f*		

	Background/ Sex	Condition	Genotype	Flies per vial	# vials	Total flies (set-up)	Deaths	Censors	Total flies (actual)	Median (d)	ΔMedian (%)		p-value (Log-Rank test)
													b
Fig. 1g	w^{Dah} female	20 mM paraquat	UAS-cat/+	10	10	100	100	0	100	0.925	0	*a*	6.9E-31
			da-GAL4>UAS-cat	10	10	100	99	1	100	3.550	+283.78	*b*	
													d
	w^{Dah} male	20 mM paraquat	UAS-cat/+	10	10	100	100	0	100	3.625	0	*c*	8.0E-16
			da-GAL4>UAS-cat	10	10	100	100	0	100	6.050	+66.90	*d*	

	Background/ Sex	Condition	Genotype	Flies per vial	# vials	Total flies (set-up)	Deaths	Censors	Total flies (actual)	Median (d)	ΔMedian (%)		p-value (Log-Rank test)	
													b	*c*
Fig. 1i	w^{Dah} female	90% O_2	UAS-cat/+	10	9	90	90	0	90	4.525	0	*a*	0.6057	1.7E-08
			da-GAL4/+	10	12	120	120	0	120	4.375	-3.31	*b*		1.4E-10
			da-GAL4>UAS-cat	10	12	120	118	2	120	4.975	+9.94	*c*		
													e	*f*
	w^{Dah} male	90% O_2	UAS-cat/+	10	12	120	119	0	119	5.475	0	*d*	0.4073	8.1E-03
			da-GAL4/+	10	12	120	110	10	120	5.400	-1.37	*e*		5.2E-04
			da-GAL4>UAS-cat	10	12	120	120	0	120	5.575	+1.83	*f*		

	Background	Sex	Genotype	Flies per vial	# vials	Total flies (set-up)	Deaths	Censors	Total flies (actual)	Median (d)	ΔMedian (%)		p-value (Log-Rank test)			
													b	*c*	*d*	*e*
Fig. S1e	w^{Dah+}	female	da-GAL4/+	15	10	150	145	3	148	57.0	0	*a*	0.8414	3.5E-07	0.0539	0.3451
			UAS-cat/+	15	10	150	149	2	151	57.3	+0.53	*b*		4.4E-08	0.0541	0.4352
			da-GAL4>UAS-cat	15	10	150	146	1	147	63.0	+10.53	*c*			9.07E-12	1.5E-09
			UAS-mito-cat/+	15	10	150	147	1	148	54.8	-3.86	*d*				0.2589
			da-GAL4>UAS-mito-cat	15	10	150	146	4	150	56.7	-0.53	*e*				
													g	*h*	*i*	*j*
Fig. S1f	w^{Dah+}	male	da-GAL4/+	20	10	200	187	9	196	61.5	0	*f*	0.6437	0.7669	0.1325	0.4905
			UAS-cat/+	20	10	200	195	6	201	61.4	-0.16	*g*		0.4550	0.2864	0.9281
			da-GAL4>UAS-cat	20	10	200	192	4	196	62.0	+0.81	*h*			0.0616	0.3259
			UAS-mito-cat/+	20	10	200	203	4	207	59.8	-2.76	*i*				0.3096
			da-GAL4>UAS-mito-cat	20	10	200	192	6	198	62.1	+0.98	*j*				

	Background/ Sex	Genotype	Condition	Flies per vial	# vials	Total flies (set-up)	Deaths	Censors	Total flies (actual)	Median (d)	ΔMedian (%)		p-value (Log-Rank test)		
													b	*c*	*d*
Fig. S1h	w^{Dah} female	da-GS>	0 µM RU	15	17	255	252	4	256	75.3	0	*a*	3.94E-12	1.26E-16	4.82E-27
		UAS-cat	50 µM RU	15	16	240	241	2	243	82.5	+9.56	*b*		0.1145	3.90E-06
			200 µM RU	15	17	255	246	5	251	83.8	+11.29	*c*			0.0016
			400 µM RU	15	17	255	215	40	255	86.7	+15.14	*d*			
													f	*g*	*h*
	w^{Dah} male	da-GS>	0 µM RU	15	17	255	239	16	255	60.9	0	*e*	0.0027	0.2355	0.4520
		UAS-cat	50 µM RU	15	17	255	247	3	250	63.1	+3.61	*f*		0.0620	0.0280
			200 µM RU	15	17	255	244	6	250	61.3	+0.66	*g*			0.7127
			400 µM RU	15	17	255	245	8	253	61.4	+0.82	*h*			

	Background	Sex	Genotype	Flies per vial	# vials	Total flies (set-up)	Deaths	Censors	Total flies (actual)	Median (d)	ΔMedian (%)		p-value (Log-Rank test)		
													b	*c*	*d*
Fig. S1i	w^{Dah}	female	+/+	10	20	200	191	8	199	72.1	0	*a*	0.7335	0.0663	3.0E-11
			UAS-cat/+	10	20	200	184	11	195	69.9	-3.05	*b*		0.0266	3.6E-12
			act5c-GAL4/+	10	20	200	192	5	197	74.3	+3.05	*c*			1.4E-06
			act5c-GAL4>UAS-cat	10	20	200	192	7	199	81.3	+12.76	*d*			

	Background	Sex	Genotype	Flies per vial	# vials	Total flies (set-up)	Deaths	Censors	Total flies (actual)	Median (d)	ΔMedian (%)		p-value (Log-Rank test)		
													b	*c*	*d*
Fig. S1j	w^{Dah+}	female	+/+	10	20	200	185	10	195	58.5	0	*a*	0.0060	0.0014	3.9E-21
			UAS-cat/+	10	20	200	191	13	204	61.7	+5.47	*b*		0.7162	4.7E-12
			da-GAL4/+	10	20	200	195	8	203	61.8	+5.64	*c*			1.5E-11
			da-GAL4>UAS-cat	10	20	200	191	5	196	70.4	+20.34	*d*			
													f	*g*	*h*
Fig. S1k	w^{Dah+}	male	+/+	10	20	200	186	10	196	52.4	0	*e*	0.1594	0.0251	0.4451
			UAS-cat/+	10	20	200	184	16	200	50.1	-4.39	*f*		0.0004	0.0477
			da-GAL4/+	10	20	200	189	9	198	56.2	+7.25	*g*			0.1916
			da-GAL4>UAS-cat	10	20	200	188	12	200	52.1	-0.57	*h*			

	Background/ Sex	Genotype	Condition	Flies per vial	# vials	Total flies (set-up)	Deaths	Censors	Total flies (actual)	Median (d)	ΔMedian (%)		p-value (Log-Rank test)			
													b	*c*	*d*	*e*
Fig. S1l	w^{Dah} female	da-GS>	–RU (0 µM)	15	18	270	255	3	258	78.0	0	*a*	2.4E-22	7.2E-08	1.0E-07	1.4E-03
		UAS-cat	+RU (200 µM) from d2	15	18	270	260	3	263	85.3	+9.36	*b*		1.7E-05	4.8E-05	8.0E-12
			+RU (200 µM) from d28	15	18	270	261	1	262	81.7	+4.74	*c*			0.8959	0.0144
			+RU (200 µM) from d42	15	18	270	250	2	252	82.0	+5.13	*d*				0.0153
			+RU (200 µM) from d56	15	18	270	261	1	262	80.9	+3.72	*e*				

p-value (Log-Rank test)

	Background	Sex	Condition	Flies per vial	# vials	Total flies (set-up)	Deaths	Censors	Total flies (actual)	Median (d)	ΔMedian (%)		*b*	*c*
**Fig. S1n**	w^{Dah}	female	UAS-cat/+	15	19	285	279	5	284	68.8	0	*a*	0.0149	0.5810
			Uo-GAL4/+	15	20	300	276	13	289	67.8	-1.45	*b*		0.0025
			Uo-GAL4>UAS-cat	15	20	300	287	7	294	69.9	+1.60	*c*		

	Background	Sex	Condition	Flies per vial	# vials	Total flies (set-up)	Deaths	Censors	Total flies (actual)	Median (d)	ΔMedian (%)		*b*	*c*
**Fig. S1o**	w^{Dah}	female	UAS-cat/+	15	19	285	279	5	284	68.8	0	*a*	0.2270	0.0023
			dilp2-GAL4/+	15	20	300	280	8	288	68.0	-1.16	*b*		3.7E-05
			dilp2-GAL4>UAS-cat	15	20	300	293	11	304	70.7	+2.76	*c*		

p-value (Log-Rank test)

	Background/ Sex	Genotype	Condition	Flies per vial	# vials	Total flies (set-up)	Deaths	Censors	Total flies (actual)	Median (d)	ΔMedian (%)		*b*
**Fig. S1p**	w^{Dah} female	elav-GS>	–RU (0 μM)	15	10	150	134	17	151	57.4	0	*a*	0.0317
		UAS-cat	+RU (200 μM)	15	10	150	148	5	153	59	+2.79	*b*	

p-value (Log-Rank test)

	Background/ Sex	Genotype	Condition	Flies per vial	# vials	Total flies (set-up)	Deaths	Censors	Total flies (actual)	Median (d)	ΔMedian (%)		*b*
**Fig. S1q**	w^{Dah} female	$S_1$106-GS>	–RU (0 μM)	15	15	225	221	5	226	65.8	0	*a*	0.0941
		UAS-cat	+RU (200 μM)	15	15	225	208	14	222	67.6	+2.74	*b*	

p-value (Log-Rank test)

	Background/ Sex	Genotype	Condition	Flies per vial	# vials	Total flies (set-up)	Deaths	Censors	Total flies (actual)	Median (d)	ΔMedian (%)		*b*
**Fig. S1r**	w^{Dah} female	TIGS2>	–RU (0 μM)	15	10	150	141	4	145	66.8	0	*a*	0.6981
		UAS-cat	+RU (200 μM)	15	10	150	148	4	152	67.4	+0.90	*b*	

p-value (Log-Rank test)

	Background/ Sex	Genotype	Condition	Flies per vial	# vials	Total flies (set-up)	Deaths	Censors	Total flies (actual)	Median (d)	ΔMedian (%)		*b*
**Fig. S1s**	w^{Dah} female	MHC-GS>	–RU (0 μM)	15	10	150	150	2	152	67.6	0	*a*	4.96E-05
		UAS-cat	+RU (200 μM)	15	10	150	139	5	144	62.1	-8.14	*b*	

p-value (Log-Rank test)

	Background/ Sex	Condition	Genotype	Flies per vial	# vials	Total flies (set-up)	Deaths	Censors	Total flies (actual)	Median (d)	ΔMedian (%)		*b*
**Fig. 2b**	w^{Dah} female	0.1x yeast	UAS-cat/+	10	16	160	157	4	161	18.25	0	*a*	3.6E-10
			da-GAL4>UAS-cat	10	16	160	158	2	160	21.45	+17.53	*b*	

p-value (Log-Rank test)

	Background/ Sex	Condition	Genotype	Flies per vial	# vials	Total flies (set-up)	Deaths	Censors	Total flies (actual)	Median (d)	ΔMedian (%)		*b* / *e*	*c* / *f*
**Fig. 2c**	w^{Dah} female	starvation (1.5% agar)	UAS-cat/+	10	8	80	80	0	80	7.050	0	*a*	0.7521	5.6E-05
			da-GAL4/+	10	12	120	120	0	120	7.125	+1.06	*b*		7.6E-07
			da-GAL4>UAS-cat	10	12	120	120	0	120	6.275	-10.99	*c*		
													e	*f*
	w^{Dah} male	starvation (1.5% agar)	UAS-cat/+	10	12	120	120	0	120	3.650	0	*d*	0.7746	0.3292
			da-GAL4/+	10	12	120	120	0	120	3.700	+1.37	*e*		0.4287
			da-GAL4>UAS-cat	10	12	120	120	0	120	3.625	-0.68	*f*		

p-value (Log-Rank test)

	Background/ Sex	Condition	Genotype	Flies per vial	# vials	Total flies (set-up)	Deaths	Censors	Total flies (actual)	Median (d)	ΔMedian (%)		*b* / *d*	*e*	*f*
**Fig. 2h**	w^{Dah+} female	da-GS>	–RU (0 μM)	15	16	240	231	10	241	81.6	0	*a*	0.4177		
		UAS-Atg5RNAi	+RU (200 μM)	15	5	225	229	2	231	79.5	-2.57	*b*			
													d	*e*	*f*
**Fig. 2i**	w^{Dah+} female	da-GS>	–RU (0 μM)	15	5	225	224	2	226	79.4	0	*c*	7.2E-15	0.1938	0.0026
		UAS-cat	+RU (200 μM)	15	5	225	224	1	225	89.0	+12.09	*d*		3.4E-09	2.0E-06
		da-GS>UAS-cat+	–RU (0 μM)	15	5	225	223	4	227	80.8	+1.76	*e*			0.1701
		UAS-Atg5RNAi	+RU (200 μM)	15	5	225	214	12	226	83.7	+5.42	*f*			

p-value (Log-Rank test)

	Background/ Sex	Condition	Genotype	Flies per vial	# vials	Total flies (set-up)	Deaths	Censors	Total flies (actual)	Median (d)	ΔMedian (%)		*b*	*c*
**Fig. S2h**	w^{Dah+} female	10 mM chloroquine	UAS-cat/+	20	8	160	147	0	147	10.4	0	*a*	0.0988	2.6E-05
			da-GAL4/+	20	8	160	156	0	156	10.8	+3.85	*b*		3.5E-09
			da-GAL4>UAS-cat	20	8	160	156	0	156	9.45	-9.13	*c*		

p-value (Log-Rank test)

	Background/ Sex	Condition	Genotype	Flies per vial	# vials	Total flies (set-up)	Deaths	Censors	Total flies (actual)	Median (d)	ΔMedian (%)		*b*	*c*	*d*
**Fig. S2j**	w^{Dah+} female	starvation (1.5% agar)	+/+	20	6	120	114	0	114	8.825	0	*a*	0.4035	0.1846	3.9E-18
			UAS-Atg5RNAi/+	20	7	140	130	0	130	8.675	-1.70	*b*		0.0297	1.6E-17
			da-GAL4/+	20	5	100	103	0	103	8.975	+1.70	*c*			7.4E-22
			da-GAL4>UAS-Atg5RNAi	20	1	20	22	0	22	7.000	-20.68	*d*			

p-value (Log-Rank test)

	Background/ Sex	Atg4a	Genotype	Flies per vial	# vials	Total flies (set-up)	Deaths	Censors	Total flies (actual)	Median (d)	ΔMedian (%)		*b* / *e*	*c* / *f*
**Fig. 4e**	w^{Dah+} female	Atg4a-WT	+/+	15	9	135	125	2	127	67.1	0	*a*	0.4857	1.8E-08
			da-GAL4/+	15	10	150	139	2	141	67.9	+1.19	*b*		6.0E-08
			da-GAL4>UAS-cat	15	10	150	144	2	146	74.5	+11.03	*c*		
													e	*f*
**Fig. 4f**	w^{Dah+} female	Atg4a-C102S	+/+	15	10	150	144	1	145	68.9	0	*d*	4.9E-04	0.2154
			da-GAL4/+	15	10	150	145	3	148	65.1	-5.52	*e*		0.0165
			da-GAL4>UAS-cat	15	10	150	141	1	142	67.8	-1.60	*f*		

p-value (Log-Rank test)

	Background/ Sex/Condition	Atg4a	Genotype	Flies per vial	# vials	Total flies (set-up)	Deaths	Censors	Total flies (actual)	Median (d)	ΔMedian (%)		*b* / *f*	*c* / *g*	*d* / *h*
**Fig. S4d**	w^{Dah+} female, 5% H_2O_2	Atg4a-WT	+/+	20	10	200	199	0	199	4.15	0	*a*	0.0836	0.0421	9.98E-95
			UAS-cat/+	20	10	200	200	0	200	4.60	10.84	*b*		0.5679	4.80E-92
			da-GAL4/+	20	8	160	149	0	149	4.25	2.41	*c*			6.19E-86
			da-GAL4>UAS-cat	20	10	200	189	0	189	11.05	166.27	*d*			
													f	*g*	*h*
	w^{Dah+} female, 5% H_2O_2	Atg4a-C102S	+/+	20	10	200	196	0	196	4.40	0	*e*	0.0094	0.3001	5.0E-93
			UAS-cat/+	20	10	200	194	0	194	4.60	+4.55	*f*		0.1531	4.8E-90
			da-GAL4/+	20	8	160	160	0	160	4.35	-1.14	*g*			3.6E-87
			da-GAL4>UAS-cat	20	10	200	199	0	199	10.75	+144.32	*h*			
