# Supplementary material for: Enhancing autophagy by redox regulation extends lifespan in Drosophila
Source: Nat Commun. 2025 Jun 25;16:5379. doi: 10.1038/s41467-025-60603-w (PMC12198390; doi:10.1038/s41467-025-60603-w)
Supplement: Supplementary file 6 — Reporting Summary [file 41467_2025_60603_MOESM6_ESM.pdf]

nature portfolio

Corresponding author(s): Linda Partridge and Helena Cochemé

Last updated by author(s): Apr 15, 2025

Reporting Summary

Nature Portfolio wishes to improve the reproducibility of the work that we publish. This form provides structure for consistency and transparency in reporting. For further information on Nature Portfolio policies, see our Editorial Policies and the Editorial Policy Checklist.

Statistics

For all statistical analyses, confirm that the following items are present in the figure legend, table legend, main text, or Methods section.

n/a	Confirmed	
☐	☒	The exact sample size (*n*) for each experimental group/condition, given as a discrete number and unit of measurement
☐	☒	A statement on whether measurements were taken from distinct samples or whether the same sample was measured repeatedly
☐	☒	The statistical test(s) used AND whether they are one- or two-sided<br>*Only common tests should be described solely by name; describe more complex techniques in the Methods section.*
☐	☒	A description of all covariates tested
☐	☒	A description of any assumptions or corrections, such as tests of normality and adjustment for multiple comparisons
☐	☒	A full description of the statistical parameters including central tendency (e.g. means) or other basic estimates (e.g. regression coefficient) AND variation (e.g. standard deviation) or associated estimates of uncertainty (e.g. confidence intervals)
☐	☒	For null hypothesis testing, the test statistic (e.g. *F*, *t*, *r*) with confidence intervals, effect sizes, degrees of freedom and *P* value noted<br>*Give P values as exact values whenever suitable.*
☒	☐	For Bayesian analysis, information on the choice of priors and Markov chain Monte Carlo settings
☒	☐	For hierarchical and complex designs, identification of the appropriate level for tests and full reporting of outcomes
☒	☐	Estimates of effect sizes (e.g. Cohen's *d*, Pearson's *r*), indicating how they were calculated

Our web collection on statistics for biologists contains articles on many of the points above.

Software and code

Policy information about availability of computer code

Data collection	QPCR data was collected with SDS2.4 software (Applied Biosystems) or the QuantStudio 7 Flex Real-Time PCR System (Thermo Scientific). Spectrophotometric molecular assays were performed on a 96-well plate reader (Fluostar Omega, BMG Labtech). Confocal images were acquired on a Leica SP5 microscope. LC-MS/MS data were acquired on an Orbitrap LTQ XL mass spectrometer (Thermo).
Data analysis	Microsoft Excel (v16) was used for general data preparation and for recording survival data. Statistical analysis was performed in GraphPad Prism (v8-10). FIJI (v2) was used for western blot and confocal image analysis. Punctae quantification was performed in CellProfiler (v4.2.8; https://cellprofiler.org). QPCR primers were designed using Primer BLAST (Ye et al., 2012). MaxQuant was used for proteomics analysis, with functional annotations obtained from DAVID (Sherman et al., 2022; https://david.ncifcrf.gov/tools.jsp). Structural modelling was perfomed using Phyre2 (Kelley et al., 2015) and visualised in Chimera X (Pettersen et al., 2021).

For manuscripts utilizing custom algorithms or software that are central to the research but not yet described in published literature, software must be made available to editors and reviewers. We strongly encourage code deposition in a community repository (e.g. GitHub). See the Nature Portfolio guidelines for submitting code & software for further information.

Data

Policy information about availability of data

All manuscripts must include a data availability statement. This statement should provide the following information, where applicable:

- Accession codes, unique identifiers, or web links for publicly available datasets
- A description of any restrictions on data availability
- For clinical datasets or third party data, please ensure that the statement adheres to our policy

The proteomics dataset has been deposited to the ProteomeXchange Consortium via the PRIDE partner repository (accession number #PXD060330, with the control condition corresponding to #PXD002195). All other data supporting the findings from this study are available within the manuscript and its Supplementary Information, or from the corresponding authors upon reasonable request. Source data are provided with this paper.

Research involving human participants, their data, or biological material

Policy information about studies with human participants or human data. See also policy information about sex, gender (identity/presentation), and sexual orientation and race, ethnicity and racism.

Reporting on sex and gender	n/a - Our study did not include human participants, human data or human biological material.
Reporting on race, ethnicity, or other socially relevant groupings	n/a - Our study did not include human participants, human data or human biological material.
Population characteristics	n/a - Our study did not include human participants, human data or human biological material.
Recruitment	n/a - Our study did not include human participants, human data or human biological material.
Ethics oversight	n/a - Our study did not include human participants, human data or human biological material.

Note that full information on the approval of the study protocol must also be provided in the manuscript.

Field-specific reporting

Please select the one below that is the best fit for your research. If you are not sure, read the appropriate sections before making your selection.

☒ Life sciences ☐ Behavioural & social sciences ☐ Ecological, evolutionary & environmental sciences

For a reference copy of the document with all sections, see nature.com/documents/nr-reporting-summary-flat.pdf

Life sciences study design

All studies must disclose on these points even when the disclosure is negative.

Sample size	Sample sizes for survival assays were based on prior published studies by our laboratories (e.g. Bjedov et al., 2010; van Dam et al., 2020; Lu at al., 2021). Full n numbers are provided in Supplementary Table 1. Sample size for proteomics followed a previously published study from our groups (Menger et al., 2015).
Data exclusions	Some flies were censored from survival assays (e.g. if accidentally stuck to the media, or squashed by the cotton plug during vial transfer). See Supplementary Table 1 for full n numbers.
Replication	The majority of lifespans (Figs. 1a,b,e, 2a,b,h,i, 4e,f and Supplementary Figs. 1j-l) and stress assays (Figs. 1f,g, 2c, 4d and Supplementary Figs. 2h,j, 4d) were repeated at least twice as independent biological experiments, except Figs. 1h,i and Supplementary Figs. 1e,f,h,i,n-s which were performed once. Full information on sample size and replication is provided in the methods section and respective figure legends.
Randomization	Samples were allocated to groups/treatments randomly. Steps were taken to reduce batch effects wherever possible, for instance experimental repeats performed independently months apart, and by different investigators.
Blinding	Experiments were performed in an un-blinded fashion, unless otherwise stated.

Reporting for specific materials, systems and methods

We require information from authors about some types of materials, experimental systems and methods used in many studies. Here, indicate whether each material, system or method listed is relevant to your study. If you are not sure if a list item applies to your research, read the appropriate section before selecting a response.

Materials & experimental systems

n/a	Involved in the study
	☒ Antibodies
☒	Eukaryotic cell lines
☒	Palaeontology and archaeology
	☒ Animals and other organisms
☒	Clinical data
☒	Dual use research of concern
☒	Plants

Methods

n/a	Involved in the study
☒	ChIP-seq
☒	Flow cytometry
☒	MRI-based neuroimaging

Antibodies

Antibodies used

The following primary antibodies were used: anti-actin (Ab1801, Ab8224, or Ab8227, AbCam), anti-Atg8 (a generous gift from K. Köhler), anti-catalase (C0979, Sigma), anti-GFP (#2955, Cell Signaling Technology).

The following secondary antibodies were used: anti-mouse IgG HRP-linked (#7076, Cell Signaling Technology), anti-mouse IgG peroxidase-conjugate (A4416, Sigma), anti-mouse IgG (H+L) Alexa Fluor 568 (A10037, Invitrogen), anti-rabbit IgG HRP-linked (#7074, Cell Signaling Technology).

Validation

anti-actin (Ab1801, AbCam): https://www.abcam.com/en-gb/products/primary-antibodies/actin-antibody-loading-control-ab1801 (cited in 232 publications)

anti-actin (Ab8224, AbCam): https://www.abcam.com/en-gb/products/primary-antibodies/beta-actin-antibody-mabcam-8224-loading-control-ab8224 (cited in 526 publications)

anti-actin (Ab8227, AbCam): https://www.abcam.com/en-gb/products/primary-antibodies/beta-actin-antibody-ab8227 (cited in 4,034 publications)

anti-Atg8 (generated and validated by Barth et al., 2011; also used in Bjedov et al. 2020)

anti-catalase (C0979, Sigma): https://www.sigmaaldrich.com/GB/en/product/sigma/c0979 (cited in 93 publications)

anti-GFP (4B10, #2955, Cell Signaling Technology): https://www.cellsignal.com/products/primary-antibodies/gfp-4b10-mouse-mab/2955 (cited in 173 publications)

Animals and other research organisms

Policy information about studies involving animals; ARRIVE guidelines recommended for reporting animal research, and Sex and Gender in Research

Laboratory animals

This study used the fruit fly, Drosophila melanogaster. Experiments were performed with white Dahomey (wDah) as the outbred parental background, either negative or positive for the endosymbiont Wolbachia, as indicated. Flies were maintained at 25ºC on a 12h:12h light/dark cycle at constant humidity (65%), and reared on sugar-yeast-agar (SYA) medium. Fly strains used were: UAS-cat (BDSC 24621), act5c-GAL4 (BDSC 4414), elav-GS (BDSC 43642), S1106-GS (BDSC 8151) obtained from the Bloomington Drosophila Stock Center; UAS-catRNAi (VDRC 6283) obtained from the Vienna Drosophila Resource Center; gstD-GFP (Sykiotis & Bohmann, 2008), UAS-Atg5RNAi (Scott et al., 2004), UAS-mito-cat (Radyuk et al., 2010), UAS-mito-QC (Lee at al., 2018), da-GAL4 (Wodarz et al., 1995), da-GS (Tricoire et al., 2009), Uro-GAL4 (Terhzaz et al., 2010), dilp2-GAL4 (Ikeya et al., 2002), TIGS-2 (Poirier et al., 2008), MHC-GS (Osterwalder et al., 2001). The Atg4a-WT control and Atg4a-C102S knock-in lines were generated by a CRISPR/Cas9 approach, as described in this study. All lines were back-crossed in the wDah background for at least 6-10 generations.

All relevant details are described in the corresponding figure legends.

Wild animals

Our study did not involve any wild animals.

Reporting on sex

Sex is clearly indicated in all figures and legends throughout.

Field-collected samples

Our study did not involve any field-collected samples.

Ethics oversight

Ethical oversight is not applicable for Drosophila work.

Note that full information on the approval of the study protocol must also be provided in the manuscript.

Plants

Seed stocks	n/a - Our study did not include plants.
Novel plant genotypes	n/a - Our study did not include plants.
Authentication	n/a - Our study did not include plants.
